# St. Mary's Hospital for Women and Children, Plaistow

**Published:** 1916-01-08

**Authors:** 


### THE DEPARTMENT OF MODERN NURSING.*

### St. Mary's Hospital for Women and Children, Plaistow.

This hospital contains twenty-nine children's cots and fifteen beds for women, and is nursed by a staff of two ward sisters, one out-patient sister, one night sister, two staff nurses, and eleven probationers. The latter are admitted for two years, but they often stay for a third year, and they generally complete their training by three years at a large general hospital. A Plaistow probationer is to be found in nearly every large London hospital, and it has happened at least once that she has been appointed sister before the end of her three years' service.

They come for one month's trial, receiving no salary, and then are paid £6 for the first year and £12 for the second. Part of the indoor uniform is provided after the first month. The hours on duty are from 7.30 A.M. to 9 P.M., with two hours off duty daily and an hour for meals; a half-day once a week, a whole day once a month, and seven weeks' holiday during the two years.

Nurses make their own beds, and dust their rooms. They live in inconvenient, insanitary quarters that have been adapted out of the adjoining old hospital building, but the money for a new home has already been raised in great part, and when it has been built the conditions under which the nurses live will be very different.

The work in the women's wards is chiefly gynæcological, but accidents and other cases are also admitted as they occur. The children are taken in at any age from birth to eleven or twelve, and the nurses take duty in turn in each ward. As far as possible, each one works for a time in the theatre, but if this is not practicable each has at least the opportunity of seeing many operations performed in the wards. The probationers also attend to out-patient work, under the senior probationer who acts as the sister. Most of them have a little experience in $x$-ray work. No massage is done, except a little "rubbing."

Lectures are given by the matron to the junior probationers, and by the resident medical officer, the former being on nursing subjects and the latter on anatomy and physiology.

The staff nurses are promoted from among the probationers if they are willing to stay, but the sisters have all been trained for three years at a general hospital.

* Previous articles: May 16, 30; June 13; July 11, 25; Aug. 8, 1914; Jan. 16, 30; Feb. 27; March 13; April 3; May 8, 22; and Oct. 9, 1915.

